# Phosphorylation-dependent regulation of ALDH1A1 by Aurora kinase A: insights on their synergistic relationship in pancreatic cancer

**DOI:** 10.1186/s12915-016-0335-5

**Published:** 2017-02-13

**Authors:** Jing Wang, Kumar Nikhil, Keith Viccaro, Lei Chang, Jacoba White, Kavita Shah

**Affiliations:** Department of Chemistry and Purdue University Center for Cancer Research, Purdue University, 560 Oval Drive, West Lafayette, IN 47907 USA

**Keywords:** Aurora kinase A, ALDH1A1, Chemical genetic, Epithelial-to-mesenchymal transition, Therapy

## Abstract

**Background:**

Epithelial-to-mesenchymal transition (EMT) and cancer stem cell (CSC) formation are key underlying causes that promote extensive metastasis, drug resistance, and tumor recurrence in highly lethal pancreatic cancer. The mechanisms leading to EMT and CSC phenotypes are not fully understood, which has hindered the development of effective targeted therapies capable of improving treatment outcomes in patients with pancreatic cancer.

**Results:**

We show a central role of Aurora kinase A (AURKA) in promoting EMT and CSC phenotypes via ALDH1A1, which was discovered as its direct substrate using an innovative chemical genetic screen. AURKA phosphorylates ALDH1A1 at three critical residues which exert a multifaceted regulation over its level, enzymatic activity, and quaternary structure. While all three phosphorylation sites contribute to its increased stability, T267 phosphorylation primarily regulates ALDH1A1 activity. AURKA-mediated phosphorylation rapidly dissociates tetrameric ALDH1A1 into a highly active monomeric species. ALDH1A1 also reciprocates and prevents AURKA degradation, thereby triggering a positive feedback activation loop which drives highly aggressive phenotypes in cancer. Phospho-resistant ALDH1A1 fully reverses EMT and CSC phenotypes, thus serving as dominant negative, which underscores the clinical significance of the AURKA-ALDH1A1 signaling axis in pancreatic cancer.

**Conclusions:**

While increased levels and activity of ALDH1A1 are hallmarks of CSCs, the underlying molecular mechanism remains unclear. We show the first phosphorylation-dependent regulation of ALDH1A1, which increases its levels and activity via AURKA. Recent global phospho-proteomic screens have revealed increased phosphorylation of ALDH1A1 at the T267 site in human cancers and healthy liver tissues where ALDH1A1 is highly expressed and active, indicating that this regulation is likely crucial both in normal and diseased states. This is also the first study to demonstrate oligomer-dependent activity of ALDH1A1, signifying that targeting its oligomerization state may be an effective therapeutic approach for counteracting its protective functions in cancer. Finally, while AURKA inhibition provides a potent tool to reduce ALDH1A1 levels and activity, the reciprocal loop between them ensures that their concurrent inhibition will be highly synergistic when inhibiting tumorigenesis, chemoresistance, and metastasis in highly aggressive pancreatic cancer and beyond.

**Electronic supplementary material:**

The online version of this article (doi:10.1186/s12915-016-0335-5) contains supplementary material, which is available to authorized users.

## Background

Pancreatic cancer is an exceptionally aggressive disease with the majority of its victims dying within 1 year of diagnosis [1]. Effective early detection and screening are not available, and tumors are typically diagnosed after metastasis. Systemic chemotherapy, radiation therapy, or a combination of both therapies is used; however, patients rapidly develop resistance, leading to a poor 5-year survival rate of only 6%. Accumulating evidence suggests that the acquisition of epithelial-to-mesenchymal transition (EMT) and cancer stem cell (CSC) phenotypes in pancreatic tumors is an important underlying cause for extensive local tumor invasion, early metastasis, drug resistance, and tumor recurrence [2]. However, the mechanisms leading to EMT and CSC phenotypes are not fully understood, which has hindered the development of effective targeted therapies and sensitive biomarkers to improve treatment outcomes in patients with pancreatic cancer.

We focused on Aurora kinase A (AURKA), as it is overexpressed in a vast majority of pancreatic tumors [3–5]. AURKA depletion inhibits in vivo tumorigenicity, enhances taxane chemosensitivity, and induces apoptosis in pancreatic cancer cells [6]. Data such as these have resulted in ongoing clinical trials of more than a dozen AURKA inhibitors. Despite these encouraging findings, AURKA remains an essential kinase required for mitosis in all dividing cells. Consequently, its inhibition in Phase II clinical trials has been associated with several adverse side effects, suggesting that collateral inhibition of AURKA in rapidly proliferating normal tissues is responsible for the undesirable side effects [7]. These findings indicate that selective therapies against oncogenic targets of AURKA exploited in cancer may be a superior option for developing effective drugs and combating collateral toxicity.

To this end, we identified aldehyde dehydrogenase 1 (ALDH1A1) as a direct substrate of AURKA using an innovative chemical genetic screen that we developed. ALDH1A1 is a pancreatic CSC marker and is highly enriched in a subpopulation of cells which are extremely resistant to chemotherapy. Furthermore, ALDH1 is highly enriched in surgical specimens from patients with pancreatic cancer who had undergone preoperative chemo-radiation therapy compared to untreated patients [8]. However, ALDH1A1 levels have not been analyzed in pancreatic cancer. As AURKA overexpression is also associated with enhanced chemoresistance, we hypothesized that AURKA may upregulate ALDH1A1 leading to EMT, CSC phenotypes, and chemoresistance in pancreatic cancer.

## Methods

Antibodies against AURKA (H-130), actin (C-2), tubulin (TU-02), and ALDH1A1 (B-5) were purchased from Santa Cruz Biotechnology (Santa Cruz, CA, USA) (for RRIDs and/or lot numbers see Additional file 1). Cell culture BxPC3, Panc1, and HEK293T cells were purchased from American Type Culture Collection (ATCC, Manassas, VA, USA). Validated antibodies against Snail (40084), Slug (40088), N-cadherin (39429), and CD44 (39435) were purchased from One World Lab (San Diego, CA, USA) and were used at 1:5000 dilution. Antibodies against vimentin (bs-0756R), E-cadherin (bs-10009R), and matrix metalloproteinase-2 (MMP-2) (bs-4599R) were purchased from Bioss Antibodies and used at 1:1000 dilution (Additional file 1). BxPC3 and Panc1 cells were cultured in RPMI with 10% fetal bovine serum (FBS) supplemented with 2 mM glutamine and antibiotics. HEK293T and Phoenix cells were cultured in Dulbecco’s modified Eagle’s medium (DMEM) with 10% FBS supplemented with 2 mM glutamine and antibiotics.

### Expression plasmids and constructs

Human influenza hemagglutinin (HA)-tagged wild-type and mutant ALDH1A1 were cloned into VIP3 mammalian vector and bacterial petDuet vector at *Eco*RI and *Xho*I sites. HA-tagged ALDH1A1 mutants were generated using site-directed mutagenesis.

### Expression and purification of AURKA, TPX2, and ALDH1A1

AURKA was prepared using the Baculovirus Bac-to-Bac Expression System according to the manufacturer’s instructions (Invitrogen). 6x-His-TPX2 and 6x-His-tagged wild-type and mutant ALDH1A1 were expressed in *Escherichia coli* and purified using the procedures previously described [9, 10].

### Transfection and retroviral infection

For generating stable cell lines, AURKA and ALDH1A1 plasmids were transiently transfected using calcium phosphate into Phoenix cells. The retroviruses were harvested and used to infect BxPC3 cells as reported previously [11].

### In vitro kinase assays

For in vitro labeling, AURKA-TPX2 complex (on Ni-NTA beads) was pre-incubated with 100 μM of ATP for 1 h in a 1× kinase buffer (50 mM Tris, 10 mM MgCl_2_) to activate AURKA. The beads were washed extensively with 1× kinase buffer to remove excess ATP, and then subjected to an in vitro kinase assay with 2 μg of 6x-His-tagged recombinant protein (wild-type or mutant ALDH1A1) in the presence of 0.5 μCi of [γ-^32^P]ATP for 15 min. Reactions were terminated upon the addition of sodium dodecyl sulfate (SDS) loading buffer and subsequently separated by SDS-PAGE gel, transferred to a polyvinylidene difluoride (PVDF) membrane, and exposed for autoradiography.

### AURKA and ALDH1A1 shRNA

AURKA short hairpin RNAs (shRNAs) were generated in our previous study [12]. Both AURKA and ALDH1A1 shRNAs were cloned into the pLKO.1 TRC vector, which was a gift from David Root [13]. The sequences are as follows:

*AURKA shRNA1 (forward):* 5′-CCGG GGC TTT GGA AGA CTT TGA AAT CTCGAG ATT TCA AAG TCT TCC AAA GCC TTTTTG-3′. *AURKA shRNA1 (reverse):* 5′- AATTCAAAAA GGC TTT GGA AGA CTT TGA AAT CTCGAG ATT TCA AAG TCT TCC AAA GCC-3′. *AURKA shRNA2 (forward):* 5′- CCGG GCA CCA CTT GGA ACA GTT TAT CTCGAG ATA AAC TGT TCC AAG TGG TGC TTTTTG-3′. *AURKA shRNA2 (reverse):* 5′-AATTCAAAAA GCA CCA CTT GGA ACA GTT TAT CTCGAG ATA AAC TGT TCC AAG TGG TGC-3′. *AURKA shRNA3 (forward):* 5′-CCGG GCC AAT GCT CAG AGA AGT ACT CTCGAG AGT ACT TCT CTG AGC ATT GGC TTTTTG-3′. *AURKA shRNA3 (reverse):* 5′-AATTCAAAAA GCC AAT GCT CAG AGA AGT ACT CTCGAG AGT ACT TCT CTG AGC ATT GGC-3′. *ALDH1A1 shRNA1 (forward):* 5′ C CGG AGC CTT CAC AGG ATC AAC AGA CTC GAG TCT GTT GAT CCT GTG AAG GCT TTT TTG 3′. *ALDH1A1 shRNA1 (reverse):* 5′ A ATT CAA AAA AGC CTT CAC AGG ATC AAC AGA CTC GAG TCT GTT GAT CCT GTG AAG GCT 3′. *ALDH1A1 shRNA2 (forward):* 5′ C CGG ACC TCA TTG AGA GTG GGA AGA CTC GAG TCT GTT GAT CCT GTG AAG GCT TTT TTG 3′. *ALDH1A1 shRNA2 (reverse):* 5′ A ATT CAA AAA ACC TCA TTG AGA GTG GGA AGA CTC GAG TCT GTT GAT CCT GTG AAG GCT 3′. Control shRNA (scrambled shRNA), AURKA, and ALDH1A1 shRNA lentiviruses were generated and used for infecting BxPC3 cells. Stable cells were generated following puromycin selection.

### Soft agar colony formation

BxPC3, Panc1, and different stable cell lines were plated in RPMI (10^3^, 10^4^, and 10^5^ cells per dish in triplicate), 0.3% agar, and 10% FBS six-well plates as reported previously [11]. Transformed colonies were counted after 3 weeks using crystal violet staining.

### Western blotting

Cells were lysed in modified radioimmunoprecipitation assay (RIPA) buffer, supplemented with protease inhibitors. Equal amounts of cell extracts were used for western blotting.

### Ubiquitylation assay

BxPC3 cells were co-transfected with ALDH1A1 or AURKA shRNA along with 6x-His-ubiquitin. After 36 h, MG132 (Sigma) was added at 10 μM final concentration for an additional 12 h. Cells were then harvested, and ubiquitylated proteins were isolated using Ni-NTA beads. The proteins were separated by SDS-PAGE and analyzed using antibodies against AURKA and ALDH1A1.

### Chemotaxis assay

Cell migration was determined using Boyden chambers as reported previously [14]. The assays were performed in replicates of three, in four independent experiments. To allow for comparison between multiple assays, the data were normalized and expressed as a percentage of the number of cells present on the membrane.

### MTT assay

Cells were seeded in 96-well plates at 5 × 10^3^ cells per 100 μL per well and cultured for 24, 48, and 72 h. The MTT assay was conducted as reported previously [15]. Experiments were repeated three times in triplicate wells to ensure reproducibility.

### Immunofluorescence

BxPC3 and Panc1 cells were grown on poly-l-lysine-coated coverslips for 24 h, fixed with 4% formaldehyde in PBS for 15 min at room temperature, and then washed three times with PBS. The cells were blocked in 1% FBS, 2% bovine serum albumin (BSA), 0.1% triton X-100 in PBS for 1 h at 25 °C. Cells were labeled with antibodies (Actin, AURKA, or ALDH1A1) for 3 h in PBS, followed by incubation with fluorescein isothiocyanate- or Texas Red-conjugated secondary antibody. Cells were visualized using a Nikon Eclipse E600 microscope (Nikon Instruments, Melville, NY, USA).

### ALDH1A1 activity assay

The ALDH1A1 activity assay was conducted in 100 mM Hepes buffer (pH 8.0) containing 2 mM DTT, 5 mM NAD^+^, 1.5 mM MgCl_2_, and 37.5 mM imidazole. The relative concentrations of wild-type and each mutant were determined by SDS-PAGE densitometry to ensure equal concentration in all samples. To measure the consequences of AURKA-mediated phosphorylation on ALDH1A1 activity, it was phosphorylated using AURKA/TPX2 in kinase buffer (37.5 mM Hepes pH 8.00, 300 μM ATP, 4 mM MgCl_2_). Each reaction was then diluted with Hepes-BSA buffer so that each sample contained 100 mM Hepes pH 8.00, 77.4 μM ATP, 1.03 mM MgCl_2_, 0.65% BSA, and 5 mM NAD^+^. Reactions were initiated upon the addition of propanal to a final concentration of 4 mM. The change in absorbance at 360 nm due to NADH formation was measured in a 96-well plate format (SpectraFluor PLUS, TECAN). The data are shown as the fractional conversion of NAD^+^ to NADH to highlight the relative intrinsic activity of wild-type and mutants and effect of AURKA-dependent phosphorylation. For the dephosphorylation experiment, ALDH1A1 activity was measured for 45 min, at which time 1 μL of calf-alkaline phosphatase was added to the sample and the measurements were performed for another 1.5 h. For untreated samples, 50% glycerol in 1x CutSmart Buffer (New England Biolabs) was added as a control.

For experiments coupling the percentage of ALDH1A1 phosphorylation with a change in its dehydrogenase activity (Fig. 3g–i), recombinant AURKA-TPX2 and ALDH1A1 (0.74 mg/mL) were reacted on a 10 μL scale in the indicated combinations in 1x kinase assay buffer containing 0.5 mM ATP at 30 °C. An identical reaction mixture for samples containing ATP was treated with a tracer amount (~0.01 μCi) of [γ-^32^P] ATP. After incubation for varying periods, the kinase reactions were terminated by adding EDTA. For activity measurements, each sample was simultaneously treated with freshly prepared 5 mM NAD^+^ and the initial reaction absorbance was recorded. Reactions were initiated upon the addition of propanal to a final concentration of 5 mM. The reaction progress curves were monitored at 360 nm every 10 min for 7 h. The reaction progress curves were plotted as (A_t_-A_o_/A_o_) to reflect the relative amounts of NADH produced over time. For measuring percent phosphate incorporated into ALDH1A1 (Fig. 3g), the radioactive reactions were terminated via trichloroacetic acid (TCA) precipitation, washed two times with 200 μL of 90% acetone, and counted. To determine the percent phosphate incorporated into ALDH1A1, the background signal from AURKA in the absence of ALDH1A1 was subtracted from that containing both ALDH1A1 and AURKA. As AURKA phosphorylates ALDH1A1 at three sites, we calculated the percentage mol phosphorylation of ALDH1A1 per mole of the protein accordingly.

### Native gel analysis of AURKA-mediated ALDH1A1 phospho-oligomeric regulation

AURKA was immunoprecipitated from BxPC3 cells, and a kinase reaction was carried out in 50 mM Tris pH 8.00, 10 mM MgCl_2_, 50 mM NaCl, and 50 mM imidazole containing ~0.3–0.6 mg/mL ALDH1A1. Notably, when high concentrations of ALDH1A1 were used (>1 mg/mL), the change from tetramer to monomer was less pronounced. The kinase reaction was initiated upon the addition of ATP to a final concentration of 1 mM. The reaction was spun down, and 10 μL of the supernatant was mixed with 5 μL of 3x native running buffer containing 10 mM EDTA for the first time point. The reaction tube was then placed on a thermomixer (800 rpm) at room temperature. 10 μL aliquots of the supernatant were collected at each time point indicated. The samples were separated using clear-native PAGE (CN-PAGE) with a 7.5% resolving gel (pH 8.9) and a 4.3% stacking gel (pH 6.7). Following electrophoresis, the gel was soaked in 1x SDS-PAGE running buffer for 5 min to denature complexes, followed by 1x transfer buffer containing 20% methanol to remove excess SDS. Proteins were transferred to a PVDF membrane, and ALDH1A1 was visualized using a monoclonal anti-6x-His antibody.

### In-gel ALDH1A1 activity assay

AURKA kinase was immunoprecipitated from BxPC3 cells and reacted for 30 min with ALDH1A1 as previously described. ALDH1A1 in the absence of AURKA was used as a control. Samples were separated using CN-PAGE 1.5 mm gels. Following electrophoresis, the gel was soaked 2x for 5 min in 100 mM Tris pH 8.8, and for an additional 5 min in 50 mL of 100 mM Tris pH 8.8 containing 15 mg nitrotetrazolium blue chloride (NBT) and 25 mg NAD^+^. Colorimetric detection of ALDH1A1 activity was carried out by incubating the gel in the same buffer as above with the addition of 1 mg of 1-methoxy phenazine methosulfate (PMS) and 5 mM benzaldehyde in the dark at 37^o^C with agitation. Bands were clearly visible after 5 min and were allowed to intensify over 45 min. The reaction was terminated by soaking the gel in a 10% acetic acid solution. The enzymatic activity was visualized as a purple band due to formazan precipitation upon NAD^+^ consumption. The gel was then fixed in 10% acetic acid and 40% methanol and stained with Coomassie G-250.

### Oligomer-specific phosphorylation of ALDH1A1

AURKA kinase was reacted with ALDH1A1 as previously described for 0, 10, and 30 min. Following CN-PAGE, the gels were fixed in 10% acetic acid and 30% methanol for 20 min and washed 3 times for 5 min with water. Phos-tag staining was carried out according to the manufacturer’s protocol. Gels were imaged using a Typhoon^©^ scanner equipped with a Phos-tag filter. Following fluorescent detection, total protein levels were visualized with Coomassie G-250 staining. Protein quantification was determined by SDS-PAGE densitometry. The Phos-tag gel was colorized using ImageJ to denote it as a phosphorylation-specific signal.

### Luciferase assay

BxPC3 cells were plated in a 96-well plate at a density of 3000 cells/well. After 12 h, the cells were transfected with 50 ng/well of Snail, Slug, CD44, or E-cadherin promoter-driven luciferase plasmids using lipofectamine (Invitrogen). Cells were simultaneously co-transfected with 50 ng/well of the pRL-SV40 Renilla luciferase plasmid (Promega) as an internal control. After 48 h, firefly and Renilla luciferase activities were measured with the Dual Luciferase kit (Thermo Fisher Scientific). The firefly luciferase signal was normalized to the Renilla luciferase signal to account for variations in transfection efficiency. All experiments were performed in triplicate, three independent times.

### Statistical analysis

Bar graph results are plotted as the average ± standard error of the mean (SEM). Significance was evaluated using a two-tailed Student’s *t* test and two-way analysis of variance and is displayed as follows: *p < 0.05, **p < 0.01, ***p < 0.001.

## Results

### ALDH1A1 is a direct target of AURKA

**Fig. 1:**
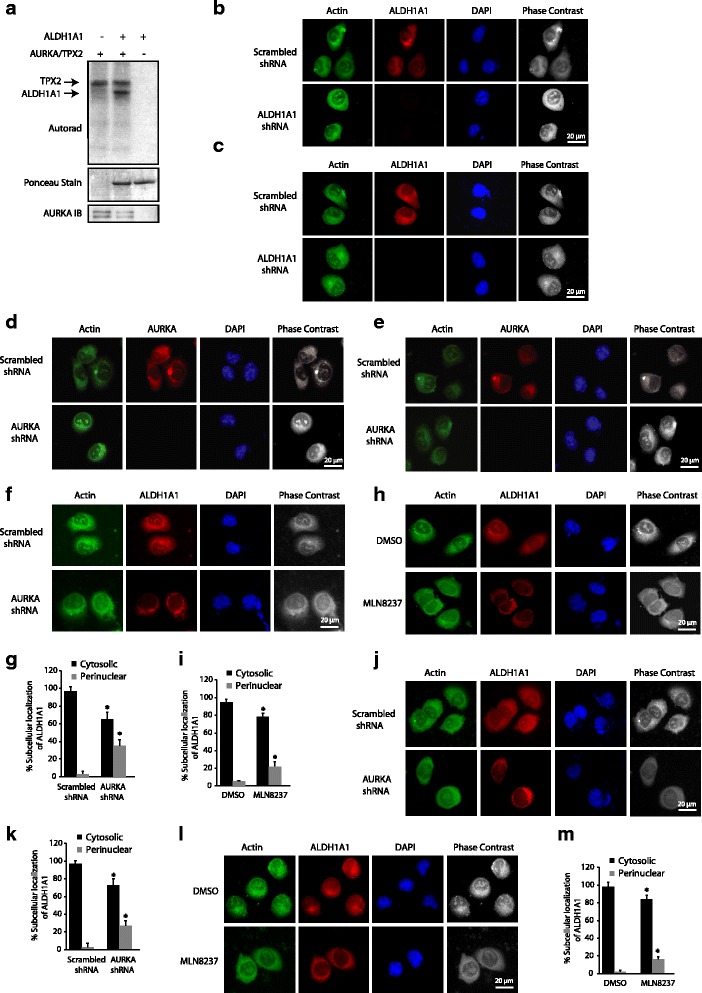
AURKA directly phosphorylates ALDH1A1 and regulates its subcellular localization. **a** ALDH1A1 is directly phosphorylated by AURKA. AURKA-TPX2 complex was subjected to kinase assay with either [^32^P]ATP (*lane 1*) or with 6x-His-ALDH1A1 and [^32^P]ATP (*lane 2*) for 15 min. *Lane 3* shows ALDH1A1 incubated with [^32^P]ATP. **b**, **c** Subcellular localization of ALDH1A1 in (**b**) BxPC3 and (**c**) Panc1 cells treated with scrambled or ALDH1A1 shRNA for 30 h. **d** AURKA localization in BxPC3 and (**e**) Panc1 cells treated with scrambled or AURKA shRNA for 30 h. **f** AURKA regulates ALDH1A1 subcellular localization in BxPC3 cells. Unsynchronized cells were either treated with scrambled (*top*) or AURKA (*bottom*) shRNA for 30 h, fixed and stained with DAPI (*blue*) or ALDH1A1 antibody (*red*). More than 100 cells were analyzed from multiple random frames. Representative data are shown. **g** Histogram shows percentage of BxPC3 cells displaying cytosolic and perinuclear localization in response to AURKA shRNA and (**i**) MLN8237. Values shown are mean ± SEM of three independent experiments. * indicate statistically significant differences with respect to controls. *p* < 0.05 analyzed by two-way analysis of variance. **h** AURKA regulates ALDH1A1 subcellular localization using its kinase activity. Unsynchronized cells were treated with either DMSO or MLN8237 for 16 h, fixed and stained with DAPI or ALDH1A1 antibody. More than 100 cells were analyzed from multiple random frames. Representative data are shown. **j**, **l** AURKA ablation (**j**) or inhibition (**l**) results in somewhat perinuclear localization of ALDH1A1 in Panc1 cells. **k**, **m** Histogram shows percentage of Panc1 cells showing cytosolic and perinuclear localization in response to (**k**) AURKA shRNA and (**m**) MLN8237. Values shown are mean ± SEM of three independent experiments. * indicate statistically significant differences with respect to controls. *p* < 0.05 analyzed by two-way analysis of variance. Data used to generate the summary statistics shown in panels **g**, **i**, **k**, and **m** are reported in Additional file 2

The chemical genetic approach involves an engineered kinase, which in the presence of a radioactive orthogonal ATP analog (e.g., *N* ^6^-(benzyl) ATP, *N* ^6^-(phenethyl) ATP), specifically transfers the radioactive tag (^32^P) to its substrates. The modified pocket in the engineered kinase is created by replacing a conserved bulky residue in the active site with glycine. The complementary substituent on ATP is created by attaching bulky groups at the N-6 position of ATP. These ATP analogs are not accepted by wild-type kinases, permitting unbiased identification of direct substrates of the engineered kinase in a global environment [9–12, 14, 16–22]. Using the aforementioned design criteria, we generated an AURKA mutant (called AURKA-as7) that efficiently accepted *N*(6)phenethyl-ATP (PE-ATP) as the orthogonal ATP analog. Using AURKA-as7 and [^32^P] PE-ATP, we previously identified several novel AURKA substrates including PHLDA1 and LIMK2 [12, 20]. In this study, we focused on ALDH1A1 as the direct target of AURKA. To confirm the results obtained from the chemical genetic screen, we conducted an in vitro kinase assay using recombinant ALDH1A1 and AURKA, which revealed that AURKA directly phosphorylates ALDH1A1 (Fig. 1a).

### AURKA regulates the subcellular localization of ALDH1A1 in pancreatic cancer cells

We next examined the subcellular localization of AURKA and ALDH1A1 in BxPC3 and Panc1 cells. ALDH1A1 displayed cytoplasmic localization, which is consistent with previous findings showing it to be a cytoplasmic enzyme (Fig. 1b and c). AURKA too displayed cytoplasmic localization in both BxPC3 and Panc1 cells (Fig. 1d and e). More importantly, when AURKA levels were knocked down using AURKA-shRNA or inhibited using MLN8237 (aka alisertib, an AURKA-specific inhibitor), ALDH1A1 adopted somewhat perinuclear localization (compare actin staining versus ALDH1A1 staining), suggesting that AURKA regulates the subcellular localization of ALDHI1A1 to some extent in BxPC3 cells (Fig. 1f and h). As shown in Fig. 1g and i, ~35% and 22% of BxPC3 cells displayed perinuclear localization of ALDH1A1 in AURKA shRNA-treated and MLN8237-treated cells, respectively. To examine whether AURKA-mediated regulation of ALDH1A1 was common in other pancreatic cancer cells, we investigated ALDH1A1 subcellular localization in Panc1 cells in the absence or presence of either AURKA shRNA or MLN8237. Similar to the results obtained in BxPC3 cells, AURKA depletion or inhibition resulted in moderate perinuclear localization of ALDH1A1 (Fig. 1j–m). Data used to generate the summary statistics shown in Fig. 1g, i, k, and m are reported in Additional file 2.

### AURKA and ALDH1A1 associate in pancreatic cancer cells

**Fig. 2:**
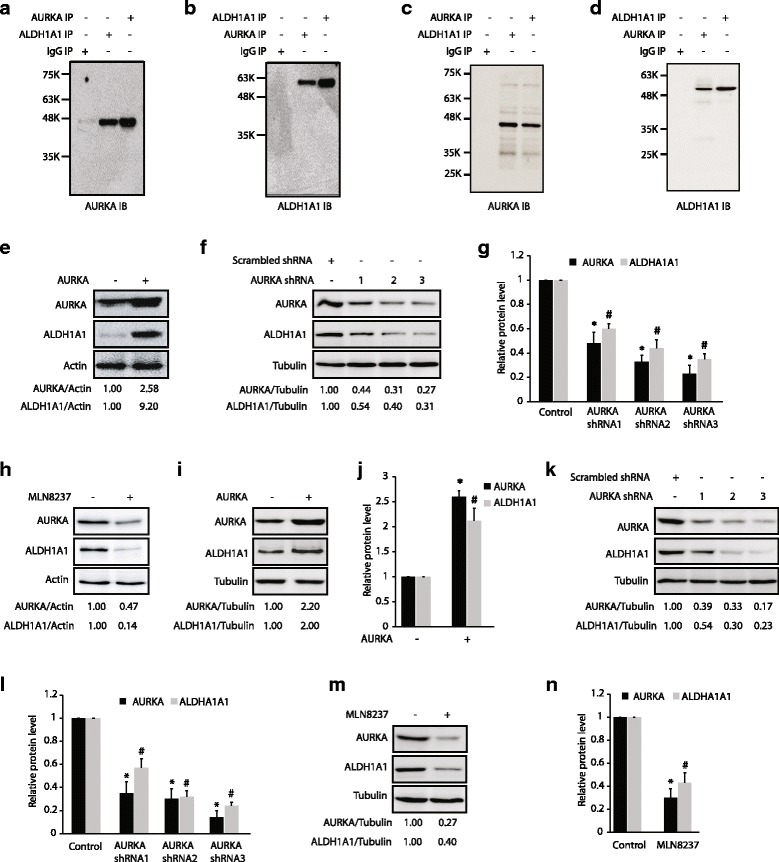
AURKA binds ALDH1A1 and positively regulates its protein levels. **a** AURKA and ALDH1A1 associate in BxPC3 cells. AURKA (*lane 3*) and IgG (*lane 1*) were used as positive and negative controls, respectively. **b** AURKA and ALDH1A1 associate in BxPC3 cells. **c**, **d** AURKA and ALDH1A1 associate in Panc1 cells, as shown by reciprocal immunoprecipitation. Each experiment was done at least three independent times. Representative data are shown. **e** AURKA overexpression increases ALDH1A1 levels. **f** AURKA ablation using three different AURKA shRNAs depletes ALDH1A1 in BxPC3 cells. **g** Histogram shows relative band intensities normalized to the corresponding tubulin level. Data are expressed as *x*-fold change relative to control; values shown as mean ± SEM of three independent experiments. * and # indicate statistically significant differences with respect to controls for AURKA and ALDH1A1 proteins, respectively. *p* < 0.05 analyzed by two-way analysis of variance. **h** AURKA inhibition using MLN8237 (1 μM for 16 h) decreases ALDH1A1 levels in BxPC3 cells. **i** AURKA overexpression increases ALDH1A1 levels in Panc1 cells. **j** Histogram shows relative band intensities normalized to the corresponding tubulin level. Results are mean ± SEM of three independent experiments. *p < 0.05 vs. AURKA control; #*p* < 0.05 vs. ALDH1A1 control analyzed by two-way analysis of variance. **k** AURKA ablation using three different AURKA shRNAs depletes ALDH1A1 in Panc1 cells. **l** Histogram shows relative band intensities normalized to the corresponding tubulin level. Results are mean ± SEM of three independent experiments. *p < 0.05 vs. AURKA control; #*p* < 0.05 vs. ALDH1A1 control analyzed by two-way analysis of variance. **m** AURKA positively regulates ALDH1A1 using its kinase activity in Panc1 cells. **n** Histogram shows relative band intensities normalized to the corresponding tubulin level. Data shown are mean ± SEM of three independent experiments. *p < 0.05 vs. AURKA control; #*p* < 0.05 vs. ALDH1A1 control analyzed by two-way analysis of variance. Data used to generate the summary statistics shown in panels **g**, **j**, **l**, and **n** are reported in Additional file 3

To investigate whether AURKA associates with ALDH1A1 in BxPC3 cells, we isolated ALDH1A1 immune complex, which pulled down AURKA (Fig. 2a). This finding was corroborated by isolating AURKA immune complex, which too revealed significant association with ALDH1A1 (Fig. 2b). Similarly, we observed robust association of AURKA and ALDH1A1 in Panc1 cells (Fig. 2c and d). These results confirm that both AURKA and ALDH1A1 associate with each other in pancreatic cancer cells.

### AURKA positively regulates ALDH1A1 levels

Our previous studies revealed that AURKA-mediated phosphorylation of PHLDA1 at S98 degrades it, whereas AURKA-mediated phosphorylation of LIMK2 at S283, T494, and T505 increases its protein stability [12, 20]. Thus, we examined whether AURKA affects the protein levels of ALDH1A1. Ectopic overexpression of AURKA increased the levels of ALDH1A1 (Fig. 2e), and its depletion using corresponding shRNAs decreased it (Fig. 2f), suggesting that AURKA positively regulates ALDH1A1 levels. Figure 2g shows quantification of alterations in ALDH1A1 levels upon AURKA knock-down from three independent experiments. We also inhibited AURKA using MLN8237, which too resulted in substantial decrease in ALDH1A1 levels, suggesting that AURKA regulates ALDH1A1 using its kinase activity (Fig. 2h). We observed similar AURKA-mediated positive regulation of ALDH1A1 in Panc1 cells, suggesting that it is a common mechanism in pancreatic cancer cells (Fig. 2i–n). Data used to generate the summary statistics shown in Fig. 2g, j, l, and n are reported in Additional file 3.

### AURKA inhibits ALDH1A1 degradation

**Fig. 3:**
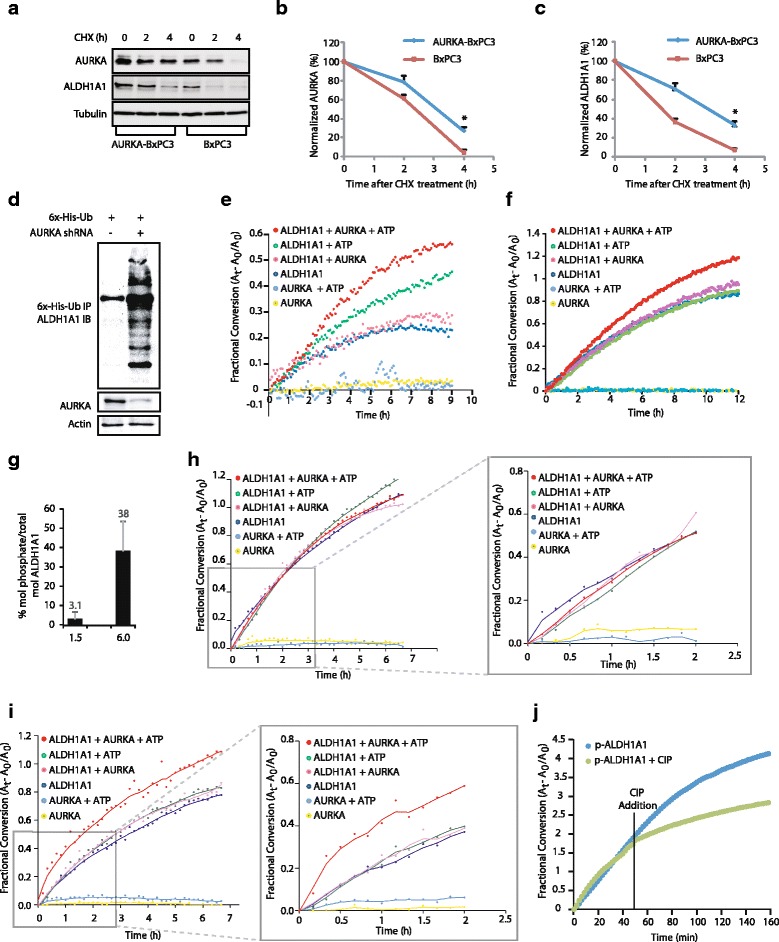
AURKA stabilizes ALDH1A1 protein levels and increases its enzymatic activity. **a** AURKA prevents ALDH1A1 degradation. AURKA-BxPC3 and BxPC3 cells were treated with cycloheximide for 2 and 4 h, and AURKA and ALDH1A1 levels analyzed. **b** Graphical representation of AURKA degradation rate. The results of densitometric scanning are shown graphically with AURKA signal normalized to actin signal. AURKA half-life is ~2 h. The significance of the difference between means was determined by Duncan’s multiple range test, *p < 0.05. **c** Graphical representation of ALDH1A1 degradation rate. ALDH1A1 half-life is ~2 h, *p < 0.05. **d** AURKA stabilizes ALDH1A1 by inhibiting its ubiquitylation. Each experiment was done at least three independent times. Representative data are shown. **e**, **f** AURKA increases ALDH1A1 enzymatic activity. Comparative spectrophotometric analysis of ALDH1A1 activity upon phosphorylation by AURKA. ALDH1A1 activity with and without ATP and AURKA were used as controls. The increase in ALDH1A1 activity observed in the presence of ATP can be overcome by stringent purification of ALDH1A1 (compare **e** and **f**). **g** Stoichiometry of ALDH1A1 phosphorylation by AURKA after 1.5 and 6 h. **h**, **i** Coupling the change in ALDH1A1 activity with stoichiometry of ALDH1A1 phosphorylation. Two identical reaction mixtures were treated with cold ATP to measure ALDH1A1 activity (**h**, **i**), or with cold ATP spiked with 0.01 μCi of [^32^P]ATP (**g**). After 1.5 and 6 h kinase reactions, ^32^P incorporation (**g**) and ALDH1A1 activity (**h**, **i**) were measured. **j** Dephosphorylation of ALDH1A1 by calf-intestinal alkaline phosphatase (*CIP*) decreases its dehydrogenase activity. Each experiment was done at least three independent times. Representative data are shown

As AURKA directly phosphorylates ALDH1A1, we hypothesized that AURKA might increase ALDH1A1 levels by inhibiting its degradation via phosphorylation. Thus, we examined the profile of ALDH1A1 degradation in BxPC3 and AURKA-overexpressing BxPC3 (AURKA-BxPC3) cells using cycloheximide. As shown in Fig. 3a–c, AURKA overexpression reduced ALDH1A1 degradation, suggesting that it regulates the level of ALDH1A1 by inhibiting its degradation. This study also revealed that the half-life of ALDH1A1 was less than 2 h in BXPC3 cells. ALDH1A1 degradation could be mediated by ubiquitin or non-ubiquitin mechanisms. We transfected 6x-His-ubiquitin into BxPC3 and AURKA-depleted-BxPC3 cells, and analyzed the ubiquitylation of ALDH1A1. Knock-down of AURKA led to increased ubiquitylation of ALDH1A1 (Fig. 3d), thus confirming that AURKA stabilizes ALDH1A1 levels by inhibiting its degradation by ubiquitylation.

### AURKA increases the enzymatic activity of ALDH1A1

Increased ALDH1A1 levels and activity are hallmarks of cancer stem cells. Thus, we investigated whether AURKA modulates the enzymatic activity of ALDH1A1. We designed a two-step approach to phosphorylate ALDH1A1 and subsequently measure its dehydrogenase activity. Due to the nature of the assay, we selected five controls to account for any phosphorylation-independent change in dehydrogenase activity. Upon phosphorylation by AURKA, we observed a robust increase in ALDH1A1 activity relative to the phosphorylated controls (Fig. 3e). During our initial experiments, we observed an increase in activity when ALDH1A1 was incubated with ATP alone; however, this change was less than that observed when in the presence of AURKA and ATP (Fig. 3e). We hypothesized that this increase was likely due to phosphorylation by bacterial kinases co-purified with ALDH1A1 and/or a direct interaction with ATP itself. We thus carried out a high-stringency purification of ALDH1A1 to eliminate potential impurities, which resulted in a robust increase in ALDH1A1 activity only when AURKA was able to phosphorylate ALDH1A1 (Fig. 3f).

We observed that the increase in ALDH1A1 activity in these experiments is highly influenced by the activity of AURKA. Accordingly, if the ratio of phosphorylated to unphosphorylated ALDH1A1 was too low, the change in activity could not be observed. Thus, we needed to address the stoichiometry of phosphorylation and correlate it with the observed increase in activity.

To answer this question, we measured the activity and the percent mol phosphate incorporated per mole of ALDH1A1 (Fig. 3g). As expected, there was virtually no observable change in ALDH1A1 activity after 1.5 h (Fig. 3h). This was not surprising, as the stoichiometry of phosphorylation was a mere 3.1% (Fig. 3g). However, after a 6-h kinase reaction, the stoichiometry of phosphorylation reached 38%, which was accompanied by an observable increase in ALDH1A1 activity (Fig. 3i). This increase was prevalent in the first several hours of the ALDH1A1 activity, as seen in the projections of each plot after 2 h (Fig. 3h, i, right panel).

As an additional approach to confirm that the increase in ALDH1A1 activity was indeed due to direct phosphorylation by AURKA, we treated phosphorylated ALDH1A1 with calf-intestinal alkaline phosphatase (CIP) and monitored the impact on dehydrogenase activity (Fig. 3j). As predicted, the addition of CIP caused a decrease in the overall activity, supporting that the change in activity observed following the kinase assay was the result of direct phosphorylation by AURKA. Combined, these findings serve to validate that ALDH1A1 enzymatic activity is regulated via phosphorylation.

### ALDH1A1 also positively regulates AURKA levels, triggering a feedback activation loop

**Fig. 4:**
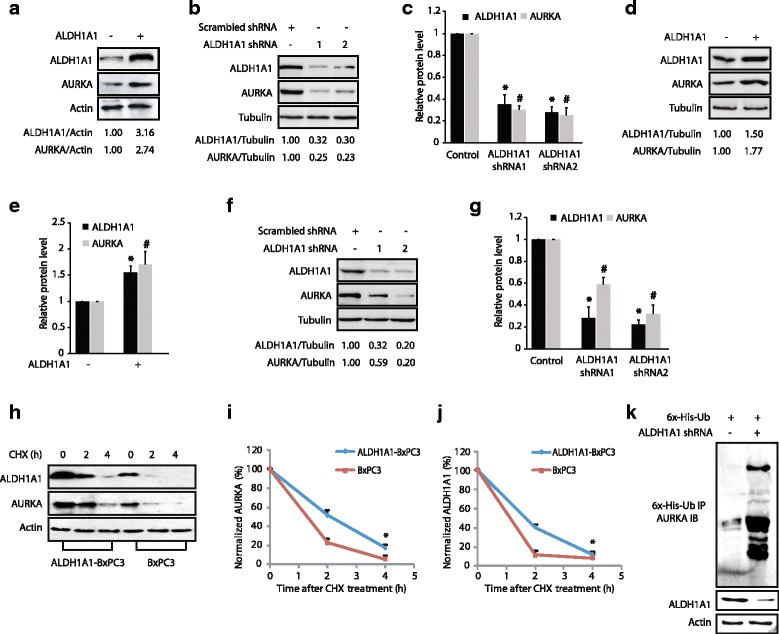
ALDH1A1 positively regulates AURKA protein levels by preventing its degradation. **a** ALDH1A1 overexpression increases AURKA levels. Wild-type HA-tagged ALDH1A1-BxPC3 cells were generated by infecting the corresponding retrovirus. **b** ALDH1A1 knock-down using two different ALDH1A1 shRNAs depletes AURKA in BxPC3 cells. Cells were transfected with scrambled shRNA (*lane 1*), ALDH1A1-shRNA1 or 2 (*lanes 2 and 3*, respectively), and AURKA and ALDH1A1 levels were analyzed after 30 h. **c** Histogram shows relative band intensities normalized to the corresponding tubulin level. Data shown are mean ± SEM of three independent experiments. * and # indicate statistically significant differences with respect to controls for ALDH1A1 and AURKA proteins, respectively. *p* < 0.05 analyzed by two-way analysis of variance. **d** ALDH1A1 overexpression increases AURKA levels in Panc1 cells. **e** Histogram shows relative band intensities normalized to the corresponding tubulin level. Data shown as mean ± SEM of three independent experiments. * and # indicate statistically significant differences with respect to controls for ALDH1A1 and AURKA proteins, respectively. *p* < 0.05 analyzed by two-way analysis of variance. **f** ALDH1A1 depletion decreases AURKA levels in Panc1 cells. **g** Histogram shows relative band intensities normalized to the corresponding tubulin level. Data shown as mean ± SEM of three independent experiments. **h** ALDH1A1 inhibits AURKA degradation. **i** Graphical representation of ALDH1A1 degradation rate. The significance of the difference between means was determined by Duncan’s multiple range test, *p < 0.05. **j** Graphical representation of AURKA degradation rate, *p < 0.05. **k** ALDH1A1 stabilizes AURKA by inhibiting its ubiquitylation. Each experiment was done at least three independent times. Representative data are shown. Data used to generate the summary statistics shown in panels **c**, **e**, and **g** are reported in Additional file 4

Interestingly, several AURKA substrates are known to regulate the levels or activity of AURKA in a feedback mechanism. Thus, we examined whether ALDH1A1 exhibits a similar impact on AURKA levels. We overexpressed ALDH1A1, which revealed a concomitant increase in AURKA levels, suggesting that a positive feedback activation loop exists between the two proteins (Fig. 4a). This finding was confirmed by depleting ALDH1A1 using two different ALDH1A1 shRNAs, which led to a robust decrease in AURKA levels (Fig. 4b). Figure 4c shows quantification of alterations in AURKA levels upon ALDH1A1 knock-down from three independent experiments. Similar results were obtained using Panc1 cells, suggesting that the AURKA-ALDH1A1 feedback activation loop is a common mechanism in pancreatic cancer cells (Fig. 4d–g). Data used to generate the summary statistics shown in Fig. 4c, e, and g are reported in Additional file 4. We next examined AURKA and ALDH1A1 levels in cycloheximide-treated BxPC3 and ALDH1A1-overexpressing BxPC3 cells. ALDH1A1 overexpression significantly reduced the degradation of AURKA, suggesting that ALDH1A1 stabilizes AURKA protein levels (Fig. 4h–j). To further corroborate this finding, we investigated the ubiquitylation of AURKA in BxPC3 and ALDH1A1-depleted BxPC3 cells, which showed increased ubiquitylation of AURKA upon ALDH1A1 depletion (Fig. 4k). Together, these results confirm that ALDH1A1 increases AURKA levels by preventing its degradation, thereby triggering a positive feedback activation loop.

### AURKA phosphorylates ALDH1A1 at T267, T442, and T493

**Fig. 5:**
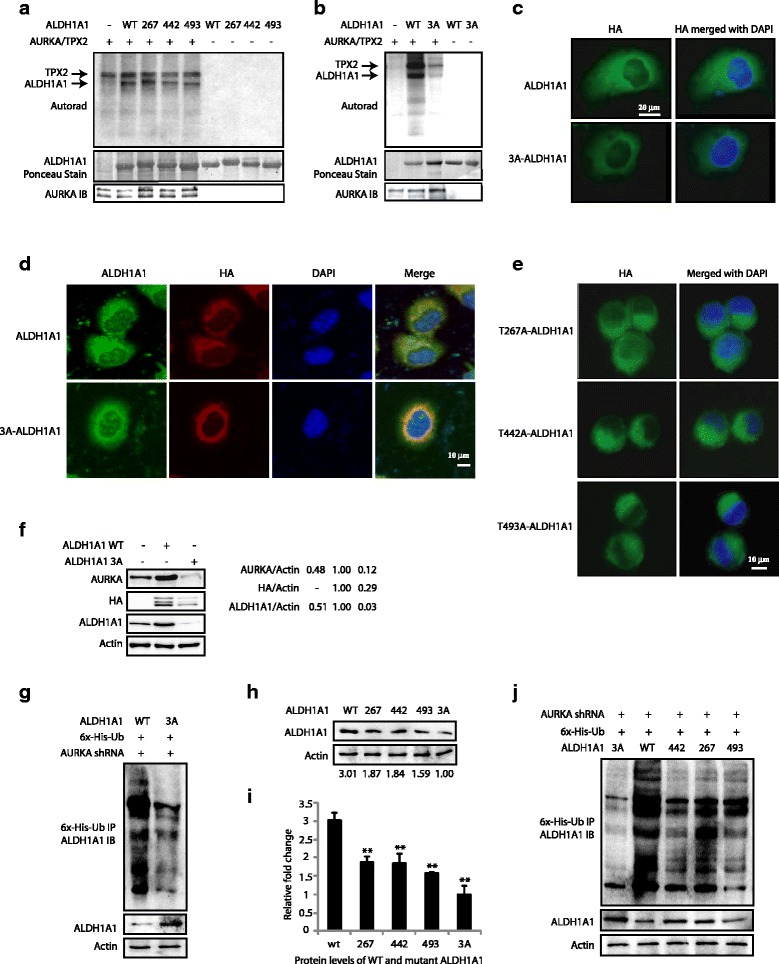
AURKA-mediated phosphorylation of ALDH1A1 at T267, T442, and T493 regulates its protein stability and subcellular localization. **a** AURKA phosphorylates ALDH1A1 at T267, T442, and T493. Phospho-dead single mutants of ALDH1A1 were subjected to an in vitro kinase assay using AURKA/TPX2 and [^32^P]ATP. **b** T267, T442, and T493 are the only AURKA sites on ALDH1A1, as the 3A-ALDH1A1 mutant is not phosphorylated by AURKA. **c** AURKA regulates the subcellular localization of ALDH1A1 via phosphorylation. HA-tagged wild-type and 3A-ALDH1A1-expressing stable cells were fixed and immunostained with DAPI (*blue*) and antibody against HA (*green*). More than 100 cells were analyzed from multiple random frames. Representative data are shown. **d** Double staining of total ALDH1A1 and ectopically expressed ALDH1A1 in wild-type and 3A-expressing BxPC3 cells. **e** AURKA regulates the subcellular localization of ALDH1A1 via phosphorylation of all three sites. More than 100 cells were analyzed from multiple random frames. Representative data are shown. **f** AURKA promotes ALDH1A1 levels by phosphorylation. BxPC3 cells were infected with HA-tagged wild-type ALDH1A1 or 3A-ALDH1A1 retrovirus. After 30 h, protein levels were analyzed using AURKA, HA, and actin antibodies. **g** AURKA inhibits ALDH1A1 ubiquitylation by phosphorylating T267, T442, and T493. **h** AURKA increases ALDH1A1 levels by phosphorylation at T267, T442, and T493. **i** Graphical representation of ALDH1A1 levels in BxPC3 cells expressing either wild-type or mutant ALDH1A1. Average values of wild-type and mutant ALDH1A1 levels from three independent experiments are depicted in the graph. **p < 0.01, compared to control analyzed by two-way analysis of variance. **j** AURKA inhibits ALDH1A1 ubiquitylation by phosphorylating T267, T442, and T493 sites. BxPC3 cells expressing either wild-type ALDH1A1, T267A-ALDH1A1, T442A-ALDH1A1, T493A-ALDH1A1, or 3A-ALDH1A1 were co-infected with AURKA shRNA along with 6x-His-ubiquitin. Ubiquitylated proteins were isolated and analyzed as described in Methods. Each experiment was done at least three independent times. Representative data are shown

AURKA preferentially phosphorylates the R/K/N-R-X-S/T-Φ consensus sequence, where Φ denotes a hydrophobic residue except for Pro [23]. This preference suggested T267, T442, and T493 as potential AURKA sites on ALDH1A1. We generated the corresponding phosphorylation-resistant mutants, T267A, T442A, and T493A and analyzed their phosphorylation using AURKA in vitro. AURKA phosphorylates all the three sites on ALDH1A1 (Fig. 5a). To investigate whether AURKA phosphorylates any additional sites, we generated the corresponding phosphorylation-resistant triple mutant (T267A, T442A, T493A, denoted as 3A) and conducted an in vitro kinase assay. AURKA did not phosphorylate the 3A mutant, confirming that T267, T442, and T493 are the only AURKA sites on ALDH1A1 (Fig. 5b).

### AURKA regulates the subcellular localization of ALDH1A1 via phosphorylation

To investigate the significance of AURKA-mediated phosphorylation of ALDH1A1, we initially investigated the subcellular localization of HA-tagged wild-type and mutant 3A-ALDH1A1 (T267A, T442A, T493A) in BxPC3 cells. While wild-type ALDH1A1 displayed cytoplasmic localization similar to the endogenous enzyme, the 3A mutant revealed perinuclear localization, showing that AURKA-mediated phosphorylation contributes to the cytoplasmic residence of ALDH1A1 (Fig. 5c). We further analyzed total ALDH1A1 (including endogenous levels) and ectopically expressed wild-type and mutant ALDH1A1 in the corresponding cell lines using ALDH1A1 and HA antibodies, respectively. While wild-type ALDH1A1-expressing cells showed diffused cytoplasmic staining of both endogenous and ectopically expressed ALDH1A1, 3A mutant-expressing cells showed distinct perinuclear staining of the 3A mutant but cytoplasmic staining for the endogenous enzyme (Fig. 5d). Together, these results underscore a role of AURKA in maintaining the cytoplasmic localization of ALDH1A1.

To investigate the contribution of each of the ALDH1A1 phosphorylation sites in regulating its subcellular localization, we generated HA-tagged wild-type and phospho-resistant single mutants of ALDH1A1-expressing BxPC3 cells, and examined their subcellular localization. Surprisingly, all single phospho-resistant mutants of ALDH1A1 showed predominantly cytoplasmic localization, suggesting that inhibition of phosphorylation at all sites is required for its perinuclear localization (Fig. 5e).

### AURKA increases ALDH1A1 levels via phosphorylation at all three sites

We next examined the consequences of AURKA-mediated phosphorylation of ALDH1A1 on its expression levels in BxPC3 cells. These cells were transfected with wild-type ALDH1A1 and the 3A-ALDH1A1 allele for 30 h, and their protein levels analyzed. As shown in Fig. 5f, while wild-type ALDH1A1 was highly expressed, 3A-ALDH1A1 was expressed at a much lower level, suggesting that AURKA-mediated phosphorylation of ALDH1A1 is required to stabilize its protein levels. Furthermore, as 3A-ALDH1A1 was expressed at a lower level, it led to a concomitant decrease in the AURKA level, presumably due to the feedback activation loop.

Our data revealed that AURKA increases ALDH1A1 levels by inhibiting its ubiquitylation; thus, we examined whether 3A-ALDH1A1 was impervious to AURKA-mediated protein stability. We transiently depleted AURKA from wild-type ALDH1A1-BxPC3 and 3A-ALDH1A1-BxPC3 cells and examined the relative ubiquitylation levels of ALDH1A1. While wild-type ALDH1A1 was significantly degraded via ubiquitylation upon AURKA depletion, 3A-ALDH1A1 showed slight ubiquitylation, confirming that AURKA-mediated phosphorylation is responsible for increased ALDH1A1 stability (Fig. 5g).

AURKA-mediated stabilization of ALDH1A1 via phosphorylation prompted us to analyze potential degron sites in the ALDH1A1 sequence, which revealed T267 within a D box motif (RVT*L). Although APC-Cdh1-mediated degradation of D-box-containing proteins can occur independently of any modification, we hypothesized that AURKA-mediated stabilization of ALDH1A1 is likely due to the phosphorylation at T267. Thus, we generated stably expressing T267A-ALDH1A1-BxPC3 cells. As controls, we also generated the other two single-mutant expressing cells (T442A-ALDH1A1-BxPC3 and T493A-ALDH1A1-BxPC3 cells). ALDH1A1 levels were analyzed in wild-type, the three single mutant expressing BxPC3 cells, and 3A-BxPC3 cells. As hypothesized, the ALDH1A1 level was reduced in T267A-ALDH1A1-BxPC3 cells compared to wild-type ALDH1A1-expressing cells; however, interestingly, it was also reduced in T442A-ALDH1A1-BxPC3 and T493A-ALDH1A1-BxPC3 cells, with the triple mutant showing minimal protein levels, suggesting that phosphorylation of each of the three sites contributes to increased ALDH1A1 levels (Fig. 5h and i).

As our data showed that AURKA increases ALDH1A1 levels by inhibiting its ubiquitylation, we analyzed the contribution of each of these three sites in affecting protein stability. The mutant ALDH1A1-expressing cell lines displayed a similar steady state decrease in ALDH1A1 levels compared to wild-type ALDH1A1-expressing cells, as shown in Fig. 5h and i. We then transiently knocked down AURKA in these cells using AURKA shRNA, which resulted in robust ubiquitylation of wild-type ALDH1A1, followed by each of the three single mutants with relatively less ubiquitylation (Fig. 5j). The 3A-ALDH1A1 mutant displayed minimal ubiquitylation upon AURKA depletion, confirming its independence from AURKA-mediated phosphorylation. These results demonstrate that AURKA-mediated phosphorylation of ALDH1A1 at each of the three sites (T267, T442, and T493) contributes to increased protein stability.

### AURKA increases ALDH1A1 activity predominantly via phosphorylation at the T267 site

**Fig. 6:**
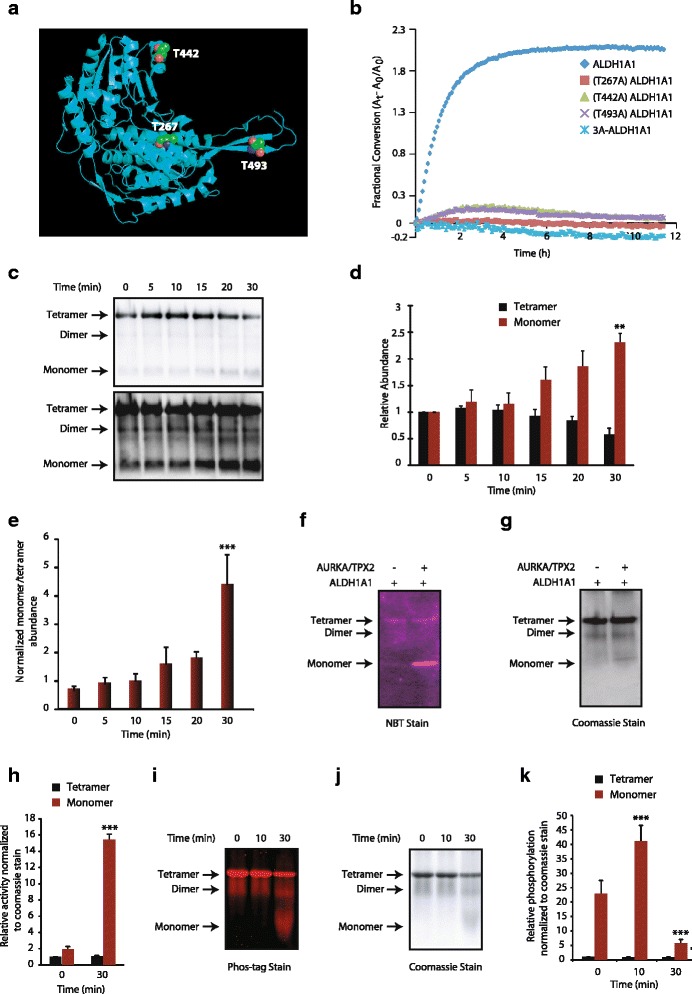
AURKA-mediated phosphorylation of ALDH1A1 modulates its oligomeric state and dehydrogenase activity. **a** Monomeric ALDH1A1 and three AURKA phosphorylation sites (4WB9). T267 is within the NAD^+^ binding pocket, T442 is within the catalytic site, and T493 is within the oligomerization domain. **b** ALDH1A1-phosphorylation-resistant mutants have diminished enzymatic activity. **c** Phosphorylation of ALDH1A1 by AURKA triggers ALDH1A1 oligomers to dissociate to the monomeric form. The same membrane is shown as a short (*top*) and long (*bottom*) exposure for clarity. **d** Average relative changes in tetramer and monomer abundance derived from three independent experiments. The intensities of tetramer and monomer at each time were normalized *independently* against time 0. Quantification of tetramer and monomer intensities was derived from the short exposure (**p < 0.01 monomer at 0 and 30 min) from three independent experiments. **e** Average ratio of normalized monomer to tetramer as a function of time. To account for the large difference in tetramer and monomer abundance, the intensity of each band was normalized against the tetramer intensity at time zero and the ratio of monomer to tetramer was plotted as a function of time. After 30 min, a significant increase in the monomer to tetramer was observed (***p < 0.01 at 0 and 30 min). **f** Activity staining of phosphorylation-induced ALDH1A1 monomer harbors high dehydrogenase activity. *Purple* color is used to denote it as activity-based staining. **g** Coomassie G-250 stain of panel **f**. **h** Quantification of oligomer-specific ALDH1A1 activities from three independent experiments (***p < 0.001 at 0 and 30 min) analyzed by two-way analysis of variance. Specifically, monomer and tetramer intensities of activity and Coomassie stain at 30 min of phosphorylation were normalized against the unphosphorylated tetramer control (basal activity), then the normalized activity of each oligomer was divided by its respective normalized Coomassie signal to generate the plot shown. **i** Phos-tag staining of phosphorylated ALDH1A1. **j** Coomassie G-250 stain of panel **i**. **k** Quantification of normalized Phos-tag intensities with respect to total protein levels from three independent experiments. Protein quantification was carried out in the same way as described for the activity assay (***p < 0.001 for monomer at 0 and 10 min and 0 and 30 min analyzed by two-way analysis of variance). The *red* color denotes it as a phosphorylation-specific signal. Data used to generate the summary statistics shown in panels **d**, **e**, **h**, and **k** are reported in Additional file 5

ALDH1A1 has an NAD^+^ binding pocket (from amino acids 8–135 and 159–270), a catalytic site (271–470) and an oligomerization domain (amino acids 140–158 and 486–495). T267 is within the NAD^+^ binding pocket (Fig. 6a). The neighboring E269 residue in the active site is essential for catalysis, suggesting that phosphorylation at T267 may have a direct impact on the catalytic activity of ALDH1A1. T442 is within the catalytic domain and T493 is part of the oligomerization domain, which is involved in oligomer formation (Fig. 6a). Thus, we hypothesized that all the three phosphorylation sites may participate in regulating ALDH1A1 enzymatic activity. Although all single mutants exhibited a robust decrease in activity relative to the wild-type enzyme, the T267 mutant showed no activity, suggesting that phosphorylation at T267 primarily governs ALDH1A1 activity (Fig. 6b).

### AURKA-mediated phosphorylation of ALDH1A1 regulates its oligomeric states

ALDH1A1 can exist in monomeric, dimeric or tetrameric forms; however, their relative activities remain unknown. As phosphorylation is known to regulate the oligomeric distribution of several proteins, we hypothesized that AURKA-mediated phosphorylation of ALDH1A1 alters its oligomeric state, and that this change was coupled with the observed change in activity. Similar to most multimeric proteins, ALDH1A1 exists predominantly as a tetramer at high concentrations; however, as the dilution increases, the equilibrium becomes more dynamic and begins to populate the monomeric form. Surprisingly, very little dimer was observed at all concentrations, suggesting that the tetramer and the monomer are its most abundant forms.

We monitored the abundance of each species upon phosphorylation as a function of time to analyze the kinetics of the phosphorylation-dependent oligomeric redistribution of ALDH1A1. To this end, we chose a concentration which contained both tetrameric and monomeric species. As the phosphorylation progressed, the ALDH1A1 oligomeric distribution drastically shifted to the monomeric form (Fig. 6c). The concomitant decrease in tetramer abundance serves as an internal control to validate that it is indeed dissociating upon phosphorylation. While Fig. 6d shows average relative changes in tetramer and monomer abundance, Fig. 6e displays the average ratio of normalized monomer to tetramer as a function of time derived from three independent experiments.

We then sought to explore which oligomeric species had the highest activity with and without phosphorylation. To this end, we conducted a similar kinetic analysis and measured the dehydrogenase activity using a native in-gel activity assay (Fig. 6f, g). Upon phosphorylation the monomeric species that formed harbored the highest activity (a greater than sevenfold increase), whereas the tetramer showed little to no change. Figure 6h shows quantification of oligomer-specific ALDH1A1 activities from three independent experiments. This is the first example to identify the oligomer-specific catalytic activities of ALDH1A1 complexes following phosphorylation.

We next sought the oligomeric species that was most phosphorylated to examine a potential correlation between phosphorylation and activity. As predicted, the monomeric population that formed was highly phosphorylated (Fig. 6i–k). Collectively, these data suggest that phosphorylation of ALDH1A1 triggers the dissociation of the tetramer into its monomeric form and that this highly phosphorylated monomer harbors the highest enzymatic activity among all of the oligomeric species. Data used to generate the summary statistics shown in Fig. 6d, e, h, and k are reported in Additional file 5.

### ALDH1A1 and AURKA feedback activation loop promotes highly aggressive pancreatic cancer phenotypes

**Fig. 7:**
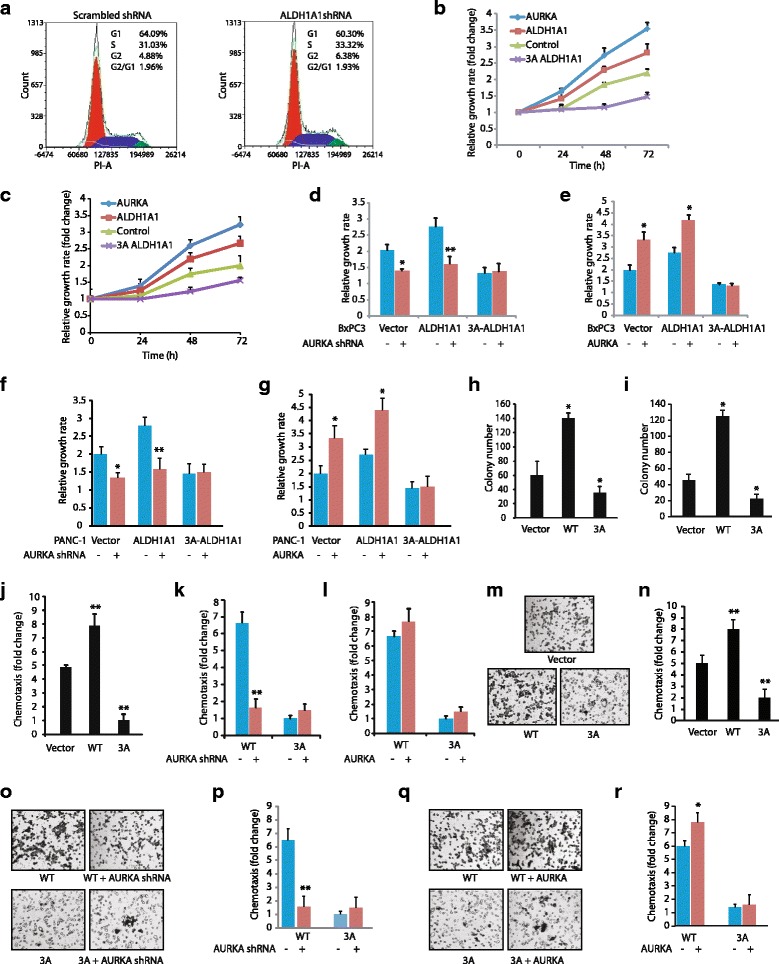
ALDH1A1 is a key oncogenic effector of AURKA. **a** scrambled shRNA-expressing BxPC3 and ALDH1A1 knock-down-BxPC3 cells were analyzed by FACS analysis. **b** ALDH1A1 promotes cell proliferation in BxPC3 cells. BxPC3, AURKA-BxPC3, ALDH1A1-BxPC3, and 3A-ALDH1A1-BxPC3 cells were plated in 96-well plates and cultured for 24, 48, and 72 h. At the end of the incubation, an MTT assay was performed. **c** ALDH1A1 promotes cell proliferation in Panc1 cells. **d** AURKA depletion decreases cell proliferation in ALDH1A1-BxPC3 cells, but not in phospho-resistant 3A-ALDH1A1-BxPC3 cells. MTT assay was performed after 48 h of transfection. The statistical significance was analyzed using two-independent-sample *t* test, *p < 0.05, **p < 0.01 compared to scrambled shRNA controls. **e** AURKA overexpression increases cell proliferation in ALDH1A1-BxPC3 cells, but not in 3A-ALDH1A1-BxPC3 cells. MTT was performed after 48 h of transfection. *p < 0.05 compared to vector-infected controls analyzed using two-independent-sample *t* test. **f** AURKA depletion decreases cell proliferation in ALDH1A1-Panc1 cells, but not in phospho-resistant 3A-ALDH1A1-Panc1 cells. **g** AURKA overexpression increases cell proliferation in ALDH1A1-Panc1 cells, but not in 3A-ALDH1A1-Panc1 cells. **h**, **i** ALDH1A1 promotes colony formation in a soft agar assay in (**h**) BxPC3 and (**i**) Panc1 cells. *p < 0.05 compared to vector-expressing control analyzed by two-way analysis of variance. **j** ALDH1A1 promotes cell motility in BxPC3 cells. **p < 0.01 compared to vector-expressing control by two-tailed Student’s *t* test. **k** AURKA depletion inhibits cell motility in ALDH1A1-BxPC3 cells, but not in phospho-resistant 3A-ALDH1A1-BxPC3 cells. **p < 0.01 compared to scrambled shRNA control by two-tailed Student’s *t* test. **l** AURKA overexpression increases cell motility in ALDH1A1-BxPC3 cells, but not in 3A-ALDH1A1-BxPC3 cells. **m** ALDH1A1 promotes cell motility in Panc1 cells. Chemotaxis assay was performed in ALDH1A1-Panc1 and 3A-ALDH1A1-Panc1 using Boyden chambers. These experiments were performed three independent times. Representative data are shown. Magnification, 200×. **n** Histogram shows mean ± SEM of three independent experiments. **p < 0.01 compared to vector-expressing control analyzed by two-way analysis of variance. **o**, **p** AURKA depletion inhibits cell motility in ALDH1A1-Panc1 cells, but not in phospho-resistant 3A-ALDH1A1-Panc1 cells. **q**, **r** AURKA overexpression increases cell motility in ALDH1A1-Panc1 cells, but not in 3A-ALDH1A1-Panc1 cells. Data used to generate the summary statistics shown in panels **c**, **f**, **g**, **i**, **n**, **p**, and **r** are reported in Additional file 6

Since AURKA is crucial for mitosis, we examined whether ALDH1A1 impacts the cell cycle. A fluorescence-activated cell sorting (FACS) analysis was conducted using unsynchronized stable scrambled shRNA-expressing BxPC3 and stable ALDH1A1-depleted BxPC3 cells. Both cell types showed a similar distribution of cells in different cell cycle phases and no aneuploidy, which suggests that ALDH1A1 does not affect the cell cycle (Fig. 7a). We next investigated the effect of AURKA-mediated phosphorylation of ALDH1A1 on cellular proliferation. As expected, ectopic expression of either AURKA or wild-type ALDH1A1 increased cellular proliferation in both BxPC3 (Fig. 7b) and Panc1 cells (Fig. 7c). In contrast, expression of 3A-ALDH1A1 showed a significantly impaired proliferation rate, which was lower than either BxPC3 (Fig. 7b) or Panc1 cells (Fig. 7c). More importantly, depletion of AURKA in BxPC3, ALDH1A1-BxPC3, and 3A-ALDH1A1-BxPC3 cells significantly reduced proliferation in ALDH1A1-BxPC3 cells but not in 3A-ALDH1A1-BxPC3 cells, suggesting that the ALDH1A1-triggered increase in cell proliferation is predominantly due to an AURKA-mediated feedback activation loop (Fig. 7d). We further tested this hypothesis by overexpressing AURKA in BxPC3, ALDH1A1-BxPC3, and 3A-ALDH1A1-BxPC3 cells. In agreement with our previous result, AURKA overexpression increased cell proliferation in wild-type ALDH1A1-BxPC3 cells, but not in 3A-ALDH1A1-BxPC3, thereby confirming that AURKA-mediated phosphorylation of ALDH1A1 is crucial for an enhanced growth rate (Fig. 7e). Similar results were obtained in Panc1 cells, where AURKA depletion significantly reduced proliferation in ALDH1A1-BxPC3 cells, but not in 3A-ALDH1A1-BxPC3 cells (Fig. 7f), whereas the AURKA overexpression mediated increase in cell proliferation in wild-type ALDH1A1-Panc1 cells, but not in 3A-ALDH1A1-Panc1 cells (Fig. 7g).

The impact of ALDH1A1 was further evaluated in BxPC3, ALDH1A1-BxPC3 and 3A-ALDH1A1-BxPC3 cells under anchorage-independent conditions. ALDH1A1 expression led to a robust increase in colony formation in BxPC3 cells compared to parental BxPC3 cells. By contrast, expression of 3A-ALDH1A1 acted as dominant negative and exhibited minimal number of colonies in the soft agar assay (Fig. 7h). Similar results were also obtained in Panc1 cells (Fig. 7i). These findings show that AURKA-mediated phosphorylation of ALDH1A1 is crucial for promoting cell proliferation both under attached and anchorage-independent conditions in pancreatic cancer cells.

### ALDH1A1 and AURKA feedback activation loop enhances cell motility

We next determined the contribution of ALDH1A1 in promoting chemotaxis using serum-starved BxPC3, ALDH1A1-BxPC3, and 3A-ALDH1A1-BxPC3 cells. A robust increase in cell motility was observed upon wild-type ALDH1A1 expression (Fig. 7j). However, expression of 3A-ALDH1A1 significantly impaired cell motility compared to vector-expressing BxPC3 cells, suggesting that 3A-ALDH1A1 may act as a dominant negative and inhibit cell motility. To further explore the impact of AURKA on ALDH1A1-mediated motility, we depleted AURKA in BxPC3, ALDH1A1-BxPC3, and 3A-ALDH1A1-BxPC3 cells, which considerably reduced cell motility in ALDH1A1-BxPC3 cells, but not in 3A-ALDH1A1-BxPC3 cells, suggesting that the ALDH1A1-triggered increase in cell motility is due to AURKA-mediated positive regulation (Fig. 7k). We further tested this hypothesis by overexpressing AURKA in BxPC3, ALDH1A1-BxPC3, and 3A-ALDH1A1-BxPC3 cells. AURKA overexpression increased cell migration in wild-type ALDH1A1-BxPC3 cells, but not in 3A-ALDH1A1-BxPC3 cells (Fig. 7l). This result was further validated in Panc1 cells, where wild-type ALDH1A1 expression showed a drastic increase in cell motility while 3A-ALDH1A1 showed significant inhibition when compared to wild-type Panc1 cells (Fig. 7m, n). Similarly, AURKA depletion significantly reduced cell motility in ALDH1A1-BxPC3 cells, but not in 3A-ALDH1A1-BxPC3 cells (Fig. 7o, p), while AURKA overexpression increased cell migration in wild-type ALDH1A1-Panc1 cells, but not in 3A-ALDH1A1-Panc1 cells (Fig. 7q, r). Data used to generate the summary statistics shown in Fig. 7c, f, g, i, n, p, and r are reported in Additional file 6. Together, these results corroborate that AURKA-mediated phosphorylation of ALDH1A1 plays a key role in increasing cell motility. As chemotaxis plays a key role in cancer metastasis, these results underscore a crucial oncogenic role of the AURKA-ALDH1A1 feedback activation loop in pancreatic malignancy.

### ALDH1A1 upregulation is a key mechanism by which AURKA increases EMT and CSC phenotypes

**Fig. 8:**
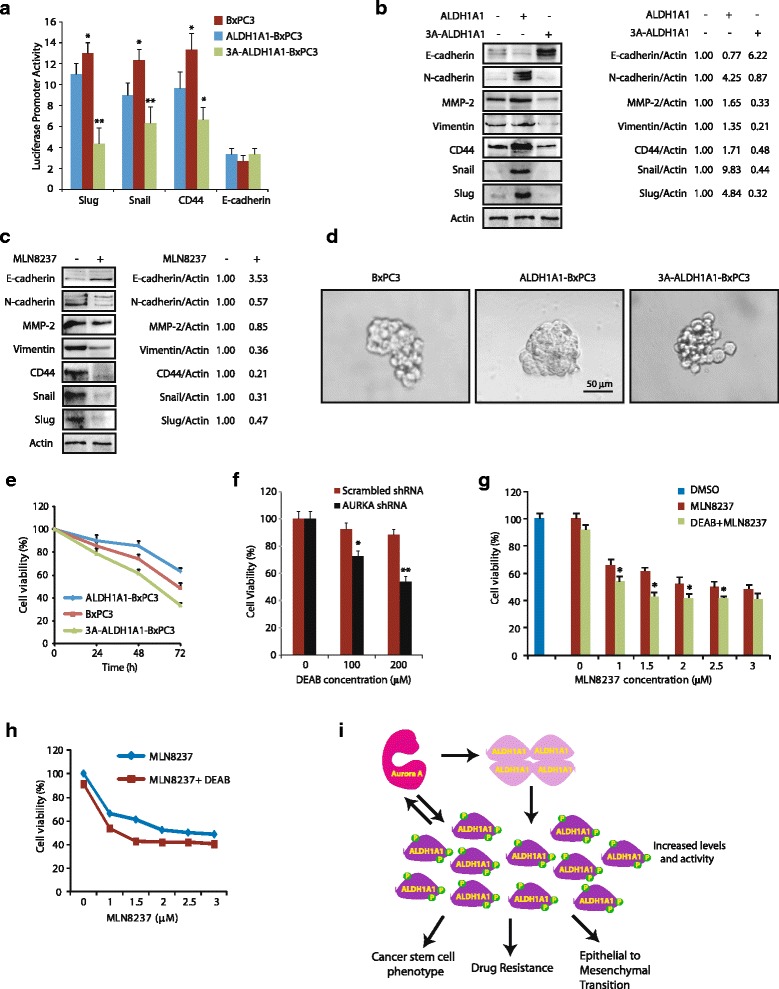
AURKA-mediated phosphorylation of ALDH1A1 is crucial for ALDH1A1-induced EMT, CSC, and drug resistance. **a** ALDH1A1 expression increases CD44, Slug, and Snail promoter activities, whereas 3A-ALDH1A1 expression decreases CD44, Slug, and Snail promoter activities. BxPC3, ALDH1A1-BxPC3, and 3A-ALDH1A1-BxPC3 cells were plated in 96-well plates overnight and then transfected with CD44, Slug, Snail, and E-cadherin promoter-driven luciferase plasmids as described in Methods. After 48 h, luciferase activities were measured with the Dual Luciferase kit (Thermo Fisher Scientific). All experiments were done in triplicate, three independent times. *p < 0.05 and **p < 0.01, compared to control BxPC3 cells analyzed by two-way analysis of variance. **b** ALDH1A1 expression increases the levels of EMT and CSC markers while decreasing E-cadherin levels, whereas 3A-ALDH1A1 expression decreases the levels of EMT and CSC markers while increasing E-cadherin levels. **c** AURKA inhibition using MLN8237 decreases the levels of EMT and CSC markers, but increases E-cadherin levels. **d** ALDH1A1 overexpression increases sphere-forming ability in BxPC3 cells. BxPC3, ALDH1A1-BxPC3, and 3A-ALDH1A1-BxPC3 cells were incubated in ultralow attachment plates as described in Methods. Pancreatospheres were counted after 5 days. **e** ALDH1A1 overexpression increases drug resistance in BxPC3 cells. BxPC3, wild-type ALDH1A1-BxPC3, and 3A-ALDH1A1-BxPC3 cells were plated in 96-well plates overnight, doxorubicin (1 μM) was added, and cells cultured for another 24, 48, or 72 h. Cell viability was calculated by MTT assay. **f** AURKA ablation sensitizes BxPC3 cells to ALDH1A1 inhibition using *N*,*N*-diethylaminobenzaldehyde (*DEAB*). BxPC3 cells were either incubated with DEAB (100 μM) or with vehicle (DMSO). After 48 h, cell viability was analyzed. *p < 0.05 and **p < 0.01 compared to scrambled shRNA control by two-tailed Student’s *t* test. **g** ALDH1A1 inhibition sensitizes BxPC3 cells to AURKA inhibition. BxPC3 cells were treated with 100 μM DEAB and varying concentrations of MLN8237 (0–3 μM) for 24 h and cell viability analyzed. **h** Same data as in **g** but shown as line graph. **i** Proposed model showing AURKA-ALDH1A1 axis in promoting EMT, CSC, and chemoresistance in pancreatic cancer

A pivotal role of ALDH1A1 in inducing EMT and CSC is well delineated. A recent study has also reported that AURKA leads to EMT using MLN8237; however, the mechanism remains unclear [24]. Our data suggest that ALDH1A1 upregulation may be a key mechanism by which AURKA leads to EMT and CSC. Thus, to test this hypothesis, we examined potential modulation of E-cadherin, Snail, Slug, and CD44p using corresponding luciferase plasmids which measure their promoter activities. As shown in Fig. 8a, expression of wild-type ALDH1A1 led to a robust increase in the activities of Snail, Slug, and CD44p, but had minimal effect on E-cadherin activity. More importantly, ectopic expression of the phosphorylation-resistant triple mutant not only prevented the increase in promoter activities of CD44, Slug, and Snail, but in effect resulted in even less activity compared to parental cells, thereby underscoring a crucial role of AURKA in ALDH1A1-induced aggressive oncogenic phenotypes.

To further confirm these results, we analyzed the protein levels of N-cadherin, CD44, Slug, Snail, and E-cadherin in BxPC3, wild-type ALDH1A1-BxPC3, and 3A-ALDH1A1-BxPC3 cells. E-cadherin is an epithelial cell marker, which is downregulated upon EMT, while Snail, Slug, and CD44p are increased in cells undergoing EMT and CSC. Similar to the promoter activation assays, N-cadherin, CD44, Slug, and Snail levels increased considerably upon ALDH1A1 expression, but were prevented in the presence of 3A-ALDH1A1 in BxPC3 cells, confirming that the phosphorylation-resistant mutant serves as a dominant negative (Fig. 8b). In addition, E-cadherin levels decreased in ALDH1A1-overexpressing cells, but showed a significant increase in 3A-ALDH1A1 cells. We also analyzed the levels of vimentin and matrix metalloproteinase-2 (MMP-2), levels of which are known to increase in EMT-undergoing cells and promote tumor invasion. ALDH1A1 overexpression increased both vimentin and MMP-2 levels in BxPC3 cells, whereas phospho-resistant mutant expression abolished their expression. Collectively, these results validate that AURKA-mediated phosphorylation plays a key role in ALDH1A1-mediated aggressive phenotypes.

In parallel, we also analyzed the levels of E-cadherin, N-cadherin, CD44, vimentin, MMP-2, Slug, and Snail in BxPC3 cells treated either with DMSO control or MLN8237. AURKA inhibition reduced the levels of N-cadherin, CD44, vimentin, MMP-2, Slug, and Snail, but increased E-cadherin levels in pancreatic cancer cell lines (Fig. 8c). These findings further underscored a key role of AURKA in promoting EMT in pancreatic cancer, presumably by modulating ALDH1A1 levels and activity.

We next conducted a sphere-forming assay to gauge the self-renewal capacity of BxPC3, ALDH1A1-BxPC3, and 3A-ALDH1A1-BxPC3 cells using ultralow attachment conditions. Under these conditions, cancer stem cells grow in suspension and form independent spheres or colonies. As shown in Fig. 8d, parental BxPC3 mainly formed aggregates of cells with no pancreatosphere formation. In contrast, overexpression of ALDH1A1 induced large pancreatosphere formation, confirming that ALDH1A1 overexpression causes the CSC phenotype. Importantly, 3A-ALDH1A1-BxPC3 cells also showed no pancreatosphere formation, thereby confirming that AURKA-mediated phosphorylation of ALDH1A1 is crucial for inducing the CSC phenotype.

### ALDH1A1 phosphorylation contributes to drug resistance

As both EMT and CSC contribute to drug resistance, we examined doxorubicin sensitivity in BxPC3 cells, which revealed about 50% loss in cell viability in 72 h at 1 μM concentration (Fig. 8e). Expression of wild-type ALDH1A1 in BxPC3 rendered these cells significantly resistant to doxorubicin (~30% loss in cell viability in 72 h), whereas 3A-ALDH1A1 expression conferred high sensitivity to doxorubicin-induced toxicity (~70% loss in cell viability) (Fig. 8e). These findings suggest that AURKA-mediated phosphorylation of ALDH1A1 considerably contributes to the drug resistance observed in patients with pancreatic ductal adenocarcinoma (PDAC). These results led us to investigate whether ablation of AURKA sensitizes BxPC3 cells to ALDH1A1 inhibition. We used *N*,*N*-diethylaminobenzaldehyde (DEAB) to inhibit ALDH1A1, although it is not very selective and inhibits other ALDH isozymes as well. As shown in Fig. 8f, AURKA-depleted cells exhibit higher sensitivity to ALDH1A1 inhibition compared to scrambled shRNA-expressing cells, suggesting that targeting the AURKA-ALDH1A1 axis is likely to be more effective than individually targeting either AURKA or ALDH1A1.

To test this hypothesis, we treated BxPC3 cells with 100 μM DEAB for 24 h, which showed minimal toxicity, suggesting inhibition of ALDH1A1 alone does not confer toxicity to cells (Fig. 8g, h, bar graph and line graph, respectively). In contrast, AURKA inhibition showed ~32% and ~35% loss in cell viability at 1 and 1.5 μM MLN8237 concentrations, respectively. More importantly, co-inhibition of ALDH1A1 and AURKA was highly effective, particularly, at 1.5 μM MLN8237 concentration, which showed more than 65% loss in cell viability. These findings underscore the significance of the AURKA-ALDH1A1 feedback activation loop in pancreatic carcinoma and suggest that concurrent inhibition of AURKA and ALDH1A1 is likely to be highly effective in targeting highly chemoresistant PDAC (Fig. 8i).

## Discussion

ALDH1A1 is a cytosolic enzyme which catalyzes the oxidation of various aldehydes including *trans*- and *cis*-retinal to their corresponding less reactive acids, thereby aiding the cell in detoxifying these compounds. ALDH1A1 also binds to endobiotics and xenobiotics and possesses antioxidant activity [25, 26]. ALDH1A1 is highly expressed in normal stem cells, but is also widely used as a marker to identify and isolate various types of CSCs. ALDH1A1 level and activity are upregulated in patients treated with neoadjuvant chemotherapy and are associated with extreme chemoresistance and poor prognosis in pancreatic cancer and beyond. Thus, there is a critical need to develop therapies that target ALDH1A1 alone or in combination to eliminate these “stem cell-like” tumor cells in pancreatic and other types of cancer.

While several studies have shown increased mRNA or protein levels of ALDH1A1 in different cancers, the molecular mechanism remains unclear. There is no report that shows phosphorylation-dependent regulation of ALDH1A1 to date. We show that AURKA directly phosphorylates ALDH1A1 at T267, T442, and T493, which stabilizes ALDH1A1. ALDH1A1 also stabilizes AURKA levels by inhibiting its ubiquitylation, thereby triggering a reciprocal feedback activation loop. Interestingly, each of the three sites contributes towards ALDH1A1 stability. Furthermore, ectopic expression of phospho-resistant ALDH1A1 prevents the promoter activation of Slug, Snail, CD44, and N-cadherin while simultaneously failing to repress E-cadherin promoter, suggesting that inhibition of AURKA-mediated phosphorylation impairs its transcriptional activity. Importantly, ectopic expression of phospho-resistant ALDH1A1 not only fully inhibits ALDH1A1-mediated oncogenic pathways, but even reduces the basal levels of Snail, Slug, N-cadherin, vimentin, MMP-2, and CD44 in BxPC3 cells, highlighting a vital role of AURKA in ALDH1A1-mediated signaling in cancer cells.

We also show that AURKA-mediated phosphorylation at the T267 site increases ALDH1A1 activity. Recent global phospho-proteomics analysis studies indeed observed increased phosphorylation at the T267 site on ALDH1A1 in human colorectal cancer and non-small lung cancer tissues [27, 28]; however, the kinase responsible remains unknown. Similarly, Han et al. reported increased phosphorylation at T267 on ALDH1A1 in human liver tissues [29]. ALDH1A1 is highly expressed in the liver and is crucial for the prevention of dietary retinol-induced liver toxicity [30], suggesting that AURKA-mediated increase in ALDH1A1 levels and activity may also be essential for normal tissues which require high ALDH1A1 activity. Thus, these studies not only underscore the clinical significance of AURKA-mediated regulation of ALDH1A1 in several cancers, but also highlight that the AURKA-ALDH1A1 axis may have an important role in normal tissue homeostasis.

Furthermore, our study showed that AURKA phosphorylates ALDH1A1 at T493, which is located within its oligomerization domain. ALDH1A1 exists as a tetramer, which is a dimer of a dimer (A-B + C-D). However, the relative enzymatic activities of its monomeric, dimeric, and tetrameric forms still remain unclear. One study reported that heating of ALDH1A1 increases the conversion of the dimeric form to the monomeric form, which is associated with decreased activity, although this study did not analyze the activity of the tetrameric form of ALDH1A1 [31]. Class II ALDH enzymes, such as ALDH2, display half-of-the-sites reactivity in tetrameric form, which can assume full reactivity depending on the pH or in the presence of divalent cations. Accordingly, increasing the Mg^2+^ concentration from 0 to 0.4 mM resulted in a two-fold increase in horse liver ALDH2 activity which was accompanied by the dissociation of tetrameric enzyme into dimers [32]. Similarly, with increasing pH, the tetrameric ALDH2 enzyme dissociated to the more active dimeric state even in the absence of Mg^2+^ ions [33]. These findings suggest that Class II ALDHs display higher activity in the dimeric state; however, it is not known whether ALDH1A1 (which belongs to Class I) displays similar kinetics in the dimeric and tetrameric states. Furthermore, the activity of a Class I ALDH such as ALDH1A1 is inhibited in the presence of Mg^2+^, suggesting that Class I and Class II ALDHs are regulated differently. To date, no study has investigated phosphorylation as a potential mechanism which governs these reported changes in ALDH1A1’s oligomeric state and enzymatic activity.

Our data show that AURKA-mediated phosphorylation of ALDH1A1 shifts the total population to its monomeric form, which harbors higher dehydrogenase activity relative to the tetramer. Surprisingly, unlike Class II ALDH, we did not observe substantial dimeric species in either the unphosphorylated or phosphorylated state. It is likely that in cells, the monomeric phosphorylated form may be re-incorporated into high order oligomers to form the active tetramer when ALDH1A1 levels are high. Alternatively, the cells may keep ALDH1A1 activity in check by storing excess enzyme in the less active tetrameric form, which is capable of readily dissociating to form the highly active phospho-monomer for rapid temporal response. While future studies are needed to uncover this mechanism, the phospho-oligomeric regulation of ALDH1A1 could be exploited as a novel therapeutic intervention.

## Conclusions

In conclusion, we unraveled a novel mechanism of ALDH1A1 regulation that is triggered by AURKA. While extreme chemoresistance and CSC formation are the defining features of pancreatic cancer, the mechanism by which AURKA may be involved in these processes has not been examined. Our discovery of ALDH1A1 as an AURKA substrate provides a novel mechanism by which AURKA promotes chemoresistance and stem cell formation in pancreatic cancer via ALDH1A1 and vice versa. Thus, targeting AURKA and ALDH1A1 in combination is expected to be highly effective in inhibiting tumorigenesis, chemoresistance, and metastasis in highly lethal pancreatic carcinoma. Furthermore, analysis of ALDH1A1 phosphorylation levels should aid in the development of diagnostic and prognostic biomarkers for identifying patients with suitable molecular phenotypes for maximal therapeutic benefit, avoiding treatment of unresponsive patients and improving overall quality of life.

## Additional files

Additional file 1: Details of the antibodies used in this study. (DOCX 14 kb)
Additional file 2: Raw data for Fig. 1g, i, k, and m. (XLSX 34 kb)
Additional file 3: Raw data for Fig. 2g, j, l, and n. (XLSX 40 kb)
Additional file 4: Raw data for Fig. 4c, e, and g. (XLSX 34 kb)
Additional file 5: Raw data for Fig. 6d, e, h, and k. (XLSX 42 kb)
Additional file 6: Raw data for Fig. 7c, f, g, i, n, p, and r. (XLSX 55 kb)

## Abbreviations

ALDH: Aldehyde dehydrogenase
ALDH1A1: Aldehyde dehydrogenase 1A1
CSC: Cancer stem cell
DEAB: *N*,*N*-diethylaminobenzaldehyde
EMT: Epithelial-to-mesenchymal transition
NBT: Nitrotetrazolium blue chloride
PMS: Phenazine methosulfate
