# Supplementary material for: Phosphorylation-dependent regulation of ALDH1A1 by Aurora kinase A: insights on their synergistic relationship in pancreatic cancer
Source: BMC Biol. 2017 Feb 13;15:10. doi: 10.1186/s12915-016-0335-5 (PMC5307883; doi:10.1186/s12915-016-0335-5)
Supplement: Additional file 1: — Details of the antibodies used in this study. (DOCX 14 kb) [file 12915_2016_335_MOESM1_ESM.docx]

|  | Antibody | Catalogue No | RRID/Lot# | Company |
| --- | --- | --- | --- | --- |
| 1 | AURKA (H-130) | SC-25425 | RRID:AB_2061345 | Santa Cruz |
| 2 | ALDH1A1 (B-5) | SC-374149 | RRID:AB_10917910 | Santa Cruz |
| 3 | α Tubulin (TU-02) | SC-8035 | RRID:AB_628408 | Santa Cruz |
| 4 | Actin (C-2) | SC-8432 | RRID:AB_626630 | Santa Cruz |
| 5 | Vimentin | SC-7558 | RRID:AB_794002 | Santa Cruz |
| 6 | MMP-2 | bs-4599R | RRID:AB_11083963 | Bioss Inc |
| 7 | E-Cadherin | bs-10009R | Lot#9L18W1 | Bioss Inc |
| 8 | N-Cadherin | PA1328 | AB_10891323 | One World Lab |
| 9 | CD44 | PA1021 | AB_10890445 | One World Lab |
| 10 | 6x-His | 00-1711-1MG | Lot# PUR01016008 | Columbia Bioscience |
| 11 | Slug | PB9439 | Lot#0941512DA863952 | Boster |
| 12 | Snail | PB9399 | Lot#0931512DA859969 | Boster |
